# Effects of electroacupuncture on perioperative anxiety and stress response in patients undergoing surgery for gastric or colorectal cancer: Study protocol for a randomized controlled trial

**DOI:** 10.3389/fpsyt.2023.1095650

**Published:** 2023-02-23

**Authors:** Yuchao Hou, Jiajing Lu, Jing Xie, Runjia Zhu, Mengdie Wu, Ke Wang, Jia Zhou, Jing Li

**Affiliations:** Yueyang Hospital of Integrated Traditional Chinese and Western Medicine, Shanghai University of Traditional Chinese Medicine, Shanghai, China

**Keywords:** perioperative anxiety, stress response, electroacupuncture, surgery, randomized controlled clinical trial (RCT)

## Abstract

**Background:**

Perioperative anxiety is one of the main psychological stresses experienced by patients who undergo cancer surgery. The surgery itself inevitably causes a stress response characterized by activation of the sympathetic nervous system and the hypothalamic–pituitary–adrenal axis. Both the perioperative anxiety and surgical stress response lead to increased levels of catecholamines and prostaglandins, which may be related to perioperative suppression of antimetastatic immunity and tumor-promoting alterations in the microenvironment. Hence, we designed this clinical trial to investigate the effect of electroacupuncture in reducing perioperative anxiety and surgical stress response.

**Methods:**

This is a randomized, single-center, parallel, and controlled clinical trial. Seventy-eight participants between the ages of 35 and 85 with gastric or colorectal cancer who plan to undergo tumorectomy will be randomly divided into an electroacupuncture group and a control group. The primary outcome will be the six-item short form of the State-Trait Anxiety Inventory score. The secondary outcomes will be the Amsterdam Preoperative Anxiety and Information Scale score; levels of plasma cortisol, adrenocorticotropic hormone, interleukin-6, and tumor necrosis factor-α; first exhaust time after surgery; postoperative quality of the recovery-15 score, numeric rating scale for pain score; and dosage of postoperative analgesics.

**Discussion:**

Cumulative studies revealed the efficacy of various types of acupuncture therapy with regard to reducing the anxiety and stress response caused by surgery. We expect that the results of this trial will provide high-quality clinical evidence for the choice of perioperative acupuncture for patients undergoing cancer surgery.

**Clinical trial registration:**

https://www.chictr.org.cn, identifier ChiCTR200003 7127.

## Introduction

Psychological factors such as anxiety are prevalent in patients undergoing surgery (1, 2). They tend to affect postoperative recovery and increase the risk of postoperative complications, hospital stays, and even mortality (3, 4). In particular, patients with cancer are naturally subject to psychological stress caused by a cancer diagnosis and prolonged treatment, including perioperative anxiety, which may trigger endocrinological and immunological responses that affect not only the short-term recovery but also the cancer progression and long-term survival rates (5).

Perioperative anxiety, which is one of the main psychological stresses experienced by patients who undergo surgery, can cause activation of the sympathetic nervous system (SNS) and the hypothalamic–pituitary–adrenal (HPA) axis. This results in the consequent release of stress hormones, including catecholamines and glucocorticoids (6). Notably, the stress response caused by surgery can similarly lead to sympathetic nerve excitation and increased secretion of stress hormones (7). Among the hormonal and metabolic changes due to the patients’ anxiety and the tissue damage itself, the increased levels of catecholamines and prostaglandins are related to perioperative suppression of antimetastatic immunity and tumor-promoting alterations in the microenvironment, which have been highlighted as key deleterious mediators of surgery (5, 8).

Presently, enhanced recovery after surgery is recommended to minimize the surgical stress response and maintain homeostasis by application of multiple interventions, including shorting-acting anesthetic agents intraoperatively and non-opioid oral analgesia for postoperative pain management (9, 10). For perioperative anxiety, anxiolytic preanesthetic medications, such as benzodiazepines, opioids, and beta-blockers, are not strongly recommended due to the adverse side effects of these drugs (11, 12). Non-pharmacological methods such as preoperative visit (13), cognitive behavioral therapy (14), music therapy (15), and acupuncture therapy (16–18) have shown potential for the alleviation of patients’ preoperative anxiety.

Acupuncture therapy as a hallmark of traditional Chinese medicine has previously been used in the field of analgesia and pain management in China, and it is now usually applied perioperatively to patients undergoing surgery, beyond the scope of traditional anesthesia (19). Moreover, acupuncture therapy appeared to be an effective approach to reduce preoperative anxiety compared with placebo or non-treatment conditions (17). Furthermore, another study investigated the application of electroacupuncture (EA) to reduce surgical stress response and found a clear reduction in the levels of stress hormones from 30 min to 10 h postoperatively in addition to the reduced postoperative anxiety (20). Based on these findings, we hypothesized that EA can reduce perioperative anxiety and postoperative stress response. This study focuses on the effects of EA on perioperative anxiety and stress response in patients undergoing surgery for gastric or colorectal cancer. The aim of this study was to provide clinical evidence for acupuncture intervention in the alleviation of perioperative anxiety and stress response. We present the protocol for this study in accordance with the SPIRIT reporting checklist.

## Methods

### Study design

**FIGURE 1:**
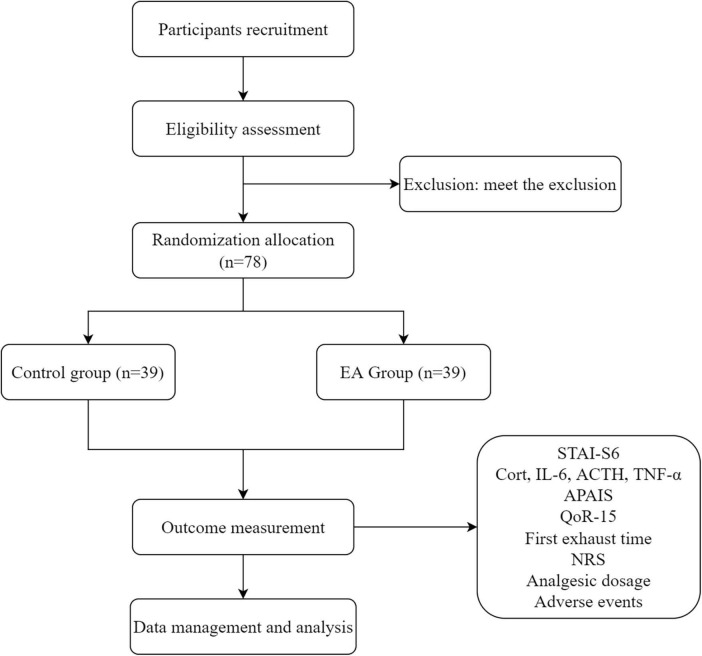
The flowchart of the trial.

This study is a randomized, single-center, parallel, and controlled clinical trial in strict accordance with the Consolidated Standards of Reporting Trials (CONSORT) and Standards for Reporting Interventions in Clinical Trials of Acupuncture (STRICTA) guidelines (21–23). This study will be conducted to evaluate the effect of EA on perioperative anxiety and stress response in patients undergoing resection of gastric or colorectal cancer. The study will be conducted in Yueyang Hospital of Integrated Traditional Chinese and Western Medicine, affiliated to Shanghai University of Traditional Chinese Medicine. A total of 78 patients will be recruited and randomly divided 1:1 into an EA group and a control group. The study design is depicted in Figure 1 and the schedule of this trial is shown in Table 1.

**TABLE 1:** The study schedule for enrollment, treatments, outcome measurements, and data collection.

Timepoint	Study period							
	**Enrollment**	**Allocation**	**Post-allocation**					
	**Pre-2**	**0**	**Pre-1**	**Pod-0**	**Pod-1**	**Pod-2**	**Pod-3**	**Pod-7**
**Enrollment**								
Inclusion criteria	×							
Exclusion criteria	×							
Informed consent	×							
Randomization	×							
Allocation		×						
**Interventions**								
EA group			×	×	×	×	×	
Control group			×	×	×	×	×	
**Outcome measurement**								
STAI-S6		×	×	×	×		×	
Cort, IL-6, ACTH, TNF-α		×			×		×	
APAIS		×	×	×				
QoR-15					×		×	
First exhaust time					×	×	×	×
NRS		×			×		×	
Analgesic dosage					×	×	×	
Adverse events			×	×	×	×	×	×

According to SPIRIT 2013 statement: defining standard protocol items for clinical trials. Pre, preoperative day; Pod, postoperative day; STAI-S6, six-item short form of the State-Trait Anxiety Inventory; Cort, cortisol; ACTH, adrenocorticotropic hormone; IL-6, interleukin-6; TNF-α, tumor necrosis factor α; APAIS, Amsterdam Preoperative Anxiety and Information Scale; QoR-15, quality of recovery-15; NRS, numeric rating scale.

### Participants

This study will include 78 participants between the ages of 35 and 85 years of age with gastric or colorectal cancer who plan to undergo tumorectomy. Based on the inclusion criteria, potential participants are being identified by screening the admission records and surgery schedule of patients in the Department of Gastroenterology Surgery.

### Inclusion criteria

Participants who fulfill the following criteria will be included: (I) 35 to 85 years of age, (II) planning to undergo surgery for gastric or colorectal cancer, and (III) willing to participate in the study and provide signed informed consent forms. There was no restriction on the inclusion of participants based on sex.

### Exclusion criteria

The exclusion criteria are as follows: (I); requiring combined removal of other organs; (II) having previously undergone abdominal surgery and presence of obvious abdominal adhesions during surgery; (III) pregnant and lactating; (IV) having anxiety, depression, other mental diseases, or cognitive dysfunction; and (V) having contraindications to acupuncture, such as local skin infection at acupoints.

### Sample size

According to the difference in the six-item short form of the State-Trait Anxiety Inventory (STAI-S6) scores between the two groups at the baseline and on the day of the surgery, the required sample size of each group will be calculated by the data statistician. Based on our previous pilot study, the difference in the standard deviation of STAI-S6 scores is 4.04 points. The STAI-S6 score changes 6.11 points in the EA group and 2.78 points in the control group. On the basis of a 5% false-positive error rate (α = 0.05, two-sided) and 80% power (β = 0.2), the sample size is estimated as follows:


$$\text{n}=\frac{2\,{\left({\text{Z}}_{\alpha /2}+{\text{Z}}_{\beta }\right)}^{2}\times \sigma^{2}}{{\left(\mu_{2}-\mu_{1}\right)}^{2}}=\frac{2\,{\left(1.96+1.28\right)}^{2}\times 4.04^{2}}{{\left(6.11-2.78\right)}^{2}}\approx 31$$

A sample size of at least 31 participants should be recruited in each group. Assuming a dropout rate of approximately 20%, a total of 78 participants will be enrolled in the trial.

### Randomization and blinding

The stratified block randomization method will be used by a researcher who is not involved in the trial. Randomization will be stratified by the surgery site (stomach or intestine) and age (<60 years or ≥60 years) and is planned to be conducted by using IBM SPSS Statistics version 25.0 software (IBM SPSS, Armonk, NY, USA). It will be performed to generate a random number table that will allocate the eligible participants to either of the two groups (the EA group or the control group) in a 1:1 ratio after the participants have signed printed informed consent forms. The random allocation cards will be made and sealed in opaque envelopes. Participants will be assigned to different groups by the Clinical Research Coordinator who will also be responsible for informing the acupuncturists regarding the group allocation. Blinding of the acupuncturists is difficult to conduct due to the characteristics of acupuncture; thus, participants and other researchers except acupuncturists will be blind to the treatment allocation including the outcome assessors and data statisticians. Participants will be arranged to different ward rooms for avoiding communicating with other participants. At the end of the last treatment, all participants will be asked to answer which group they’re allocated aiming at evaluating the success rate of blinding.

### Interventions

After signing the informed consent forms, all the eligible participants will be randomized into one of the two groups (the EA group or the control group). Participants of the EA group will receive six sessions of EA starting from 1 day before the surgery to the third day after the surgery. On the day of surgery, the EA session will be performed 30 min before the surgery until the surgery ends; while on all other days, the EA session will be performed in the afternoon. All acupuncture sessions will be delivered by experienced acupuncturists according to the standard operating procedures at the acupoints as follows: DU23 (Shangxing), DU20 (Baihui), DU29 (Yintang), LI4 (Hegu), PC6 (Neiguan), LU9 (Taiyuan), ST36 (Zusanli), ST37 (Shangjuxu), and LR3 (Taichong), all of which are selected based on the previous systematic review and our clinical experience (17). Disposable sterile stainless steel acupuncture needles (40 mm in length and 0.25 mm in diameter) will be inserted at the acupoints to a depth of 10–20 mm, which will be determined by the acupuncturists according to the participants’ body type. De qi sensation will be obtained through rotating the needles, and then the needles on the DU23 (Shangxing), DU29 (Yintang), PC6 (Neiguan), and LU9 (Taiyuan) acupoints will be stimulated using the HANS acupoint nerve stimulator (HANS-200A; Nanjing Jisheng Medical Co) at a constant frequency of 2 Hz and an intensity of 5 mA for 30 min per session. Participants assigned to the control group will receive the same course of treatment as the EA group. However, the acupuncturists will withdraw the needles immediately without De qi sensation, and the EA device will be connected to the acupoints by insulating tapes without the current passing.

### Outcome measurement

All the outcome measurements will be performed by an independent researcher who will be blinded to the group allocation.

### Primary outcome measures

The primary outcomes will measure changes in the STAI-S6 scores between the baseline and on the day of the surgery. The STAI-S6 will be used to assess the anxiety levels during the perioperative period. It is a standardized short form of the Spielberger STAI, in which the 40 questions of the Spielberger STAI have been reduced to three anxiety-present and three anxiety-absent questions (24, 25). Because of its simplicity, the STAI-S6 can be completed rapidly by participants, and it has been used to assess anxiety levels, including surgery-related anxiety levels, in many previous studies (16, 26).

### Secondary outcome measures

Besides the primary outcome measurement, the STAI-S6 will be assessed 1 day before the surgery, 1 day after the surgery, and 3 days after the surgery as well, aiming to observe the anxiety levels during the perioperative period. Assessment for the each group at other time points except the baseline will be conducted following 30 min of the intervention.

The stress response will be evaluated by measurement of the levels of plasma cortisol (Cort), adrenocorticotropic hormone (ACTH), interleukin-6 (IL-6), and tumor necrosis factor-α (TNF-α). The stress response to surgery is characterized by activation of the HPA axis and the SNS, in which ACTH synthesized by the anterior pituitary and Cort secreted by the adrenal cortex are the two representative hormones that increase in response to a surgical stimulus (7). It is well established that IL-6 and TNF-α participate in the activation of the HPA axis and increase plasma concentrations of ACTH and Cort (27). The levels of both of these cytokines have been measured in previous studies to evaluate the intensity of surgery-related stress response (28, 29). The levels of plasma Cort, ACTH, IL-6, and TNF-α will be measured 1 day before the surgery and 1 and 3 days after the surgery. To avoid the influence of circadian fluctuation, the plasma samples will be obtained strictly at the same time in the morning.

The Amsterdam Preoperative Anxiety and Information Scale (APAIS) will be used to specifically assess the preoperative anxiety levels. This scale consists of four questions related to anxiety and has been proven to correlate well with the full version of the STAI (30). The Chinese version of the APAIS has a high degree of reliability and validity for the Chinese population in the evaluation of preoperative anxiety (31). The APAIS will be assessed at the baseline, 1 day before the surgery, and on the day of the surgery.

To evaluate recovery, the first exhaust time after surgery and postoperative quality of recovery (QoR)-15 scores will be used. The first exhaust time has been frequently used in many clinical trials for assessing postoperative gastrointestinal function recovery (32, 33). The QoR-15 is a patient-reported outcome questionnaire that focuses on the quality of recovery after surgery and anesthesia (34). It has been widely used in surgery-related clinical trials as a measurement instrument for postoperative quality of recovery, and both the English and Chinese versions have been shown to have good content validity and internal consistency (35). The QoR-15 will be assessed 1 and 3 days after the surgery.

The postoperative pain will be evaluated using the numeric rating scale for pain, which is a single 11-point numeric scale with 0 representing “no pain” and 10 representing “worst pain imaginable” (36). All participants will be asked to report pain intensity in the last 24 h on the first, second, and third day after the surgery. Dosage of postoperative analgesics will also be recorded as an indicator of the pain condition.

### Adverse events and safety

Routine blood, renal function, and liver function tests will be conducted during the trial to ensure the safety of the participants. Adverse events including postoperative adverse reactions, such as infection, nausea, and vomiting, as well as potential adverse events related to acupuncture, such as palpitation, fainting, and bleeding, that occur from the time of signing the informed consent form till the last follow-up will be recorded. Regardless of a causal relationship with the study intervention, any adverse medical reaction that occurs will be evaluated and immediately reported to the primary investigator and ethics committee to decide if the patient needs to withdraw from the trial.

### Data collection and management

Screeners will collect the demographic and baseline characteristic data when the patients are recruited. Outcomes assessors will evaluate the clinical outcomes from 1 day before the surgery to 3 days after the surgery. The anxiety-related questionnaires will be completed by the subjects independently in the ward where outcome assessors will be available to answer questions. To maintain the participants’ confidentiality, only personal and study-related information will be collected and stored with restricted access. A data administrator will be responsible for cleaning, identifying, encoding, and converting the initial data to the appropriate format for data analysis. Subjects may withdraw from the study irrespective of the timing and reasons. The withdrawal data and reasons for withdrawal will be recorded. A data monitoring committee composed of independent researchers will monitor the study.

### Statistical analysis

The statistical analysis of all data will be performed by two analytical researchers, independent of the trial. All the outcomes will be subjected to an Intent-to-Treat analysis based on the initial allocation. For missing data, the multiple imputation method will be applied. IBM SPSS Statistics (version 25.0) will be used for statistical analysis. All continuous variables conforming to normal distribution will be expressed as mean ± standard deviation, while those that do not conform to normal distribution will be expressed as median and quartile spacing. For enumerative variables, frequency and the corresponding percentage will be provided. To compare variables between the two groups, independent samples *t*-test for normally distributed data and the nonparametric Mann–Whitney *U* test for non-normally distributed data will be used. Different time point assessments will be analyzed using generalized estimating equation. Differences within groups will be assessed using the paired *t*-test for normally distributed data and the Wilcoxon signed-rank test for non-normally distributed data.

## Discussion

In this randomized controlled trial of patients undergoing surgery for gastric or colorectal cancer, we intend to investigate the effect of EA on perioperative anxiety and stress response in this group of patients. Our first aim is to determine whether EA applied during the perioperative period has an antianxiety effect for patients undergoing gastric or colorectal cancer surgery. Acupuncture has been investigated as a non-pharmacological method for reducing anxiety. Several cumulative studies revealed the efficacy of various types of acupuncture therapy with regard to the reduction of preoperative anxiety (17, 37–42). Wiles et al. conducted a trial and concluded that acupuncture at the EX-HN3 point can reduce preoperative anxiety levels in neurosurgical patients immediately before surgery (16). To date, none of these studies included patients undergoing cancer surgery, which is a group of patients that appears to be more vulnerable to increased levels of circulating catecholamine trigged by perioperative anxiety (8). The main principle of the EA treatment in this trial is regulating the qi and tranquilizing the mind. Notably, DU23 and LU9 are used to tranquilizing the mind as they belong to “13 gui acupoints” based on TCM theroy. DU20 and DU29 are also used for the mind and commonly chosen in clinical practice for anxiety disorders. LI4, PC6, ST36, ST37, and LR3 are used to regulating the qi of the patients undergoing abdominal surgery for cancer.

Our second aim is to investigate the effect of EA on the surgical stress response. Increased neural signaling in the SNS and HPA axis results in increased levels of circulating stress hormones, such as glucocorticoids and catecholamines, which act on both tumor cells and tumor microenvironment, thereby inducing immunosuppression and activating β-adrenoceptors to support the development of metastasis and cancer recurrence (43–45). Therefore, it is important to seek available interventions that can reduce the surgical stress response. Previous studies revealed that acupuncture may activate the neuroendocrine system regulating corticotrophin-releasing hormone and prolactin *via* β-endorphin (46, 47). Moreover, Dalamagka et al. reported that low-frequency EA applied perioperatively exhibited greater reductions in Cort and ACTH levels at 30 min, 90 min, and 10 h postoperatively than those in the sham control group (20). In this study, the levels of plasma Cort, ACTH, IL-6, and TNF-α will be measured 1 and 3 days after the surgery to provide more evidence for the effect of acupuncture in reducing the surgical stress response.

In summary, this study is the first trial to use electroacupuncture as an intervention reduce perioperative anxiety and stress response in patients undergoing cancer surgery. The goal of this study is to evaluate the effectiveness of electroacupuncture compared with sham acupuncture including minimally penetrating. Our findings will add to the available literature on reducing the anxiety during the perioperative period and will further explain the application of perioperative acupuncture. We expect that the findings of this trial will provide robust scientific and clinical evidence for the choice of perioperative acupuncture for patients undergoing cancer surgery.

The primary limitation of our trial is that the intervention received in control group also includes the puncture of needles into the skin which may slightly stimulate the acupoint. A fMRI study demonstrated that bodily attention activates the salience network and deactivate the default mode network regardless of stimulation type (48). Some evidence revealed that EA stimulation could cause much more activation in brain regions than manual acupuncture stimulation (49). To some extent, the placebo control in this trial may underestimate acupuncture’s treatment effect while satisfying blindness and minimizing the nocebo effect. Second, the acupuncturist will not blind in this study due to the implementability. Also, as a pilot trial, this study has been designed to conduct in one single center with a relatively small sample size.

## Trial status

The protocal version is 2.1 (in 27 August 2020). The clinical trial has been planned to start on 1 October 2020 and be completed on 30 September 2022. However, due to the impact of COVID-19 pandemic, the case recruitment is undergoing at present.

## Ethics statement

The studies involving human participants were reviewed and approved by the Ethics Committee of Yueyang Hospital of Integrated Traditional Chinese and Western Medicine. The patients/participants provided their written informed consent to participate in this study.

## Author contributions

JZ and JL: conception and design. JZ: administrative support. MW and JX: provision of study materials or patients. YH and JL: collection and assembly of data. KW: data analysis and interpretation. YH, JL, JX, RZ, MW, KW, JZ, and JL: manuscript writing and final approval of manuscript. All authors contributed to the article and approved the submitted version.
